# An EEG-network-metric based approach to real-time trust inference in human-autonomy teaming

**DOI:** 10.3389/fnrgo.2025.1627483

**Published:** 2025-09-23

**Authors:** Gregory Bales, Allison P. A. Hayman, Torin K. Clark, Jason Dekarske, Sanjay Joshi, Zhaodan Kong

**Affiliations:** ^1^Cyber-Human-Physical Systems Lab, Department of Mechanical and Aerospace Engineering, University of California, Davis, Davis, CA, United States; ^2^Ann and H.J. Smead Aerospace Engineering Sciences, College of Engineering and Applied Science, University of Colorado, Boulder, CO, United States

**Keywords:** human-robot interaction, human-autonomy teaming, autonomous robots, intelligent automation, cognitive robotics, electroencephalography, cognitive processes, network theory

## Abstract

Efficient and effective teaming between humans and autonomous systems requires the establishment and maintenance of trust to maximize team task performance. Despite advances in autonomous systems, human expertise remains critical in tasks fraught with deviations from procedures or plans that cannot be pre-programmed. As autonomous systems become more sophisticated, they will possess the ability to positively influence interactions with their human partners, provided the autonomous systems have a real-time estimation of their human partner's cognitive state (including trust). In this paper, we report our results in ascertaining a human's trust in an autonomous system via electroencephalogram (EEG) measurements. We report that trust can be measured continuously and unobtrusively, and that using analysis techniques which account for interactions among brain regions shows benefits compared to more traditional methods which use only EEG signal-power. Inter-channel connectivity network-metrics, which measure dynamic changes in synchronous behavior between distant brain regions, appear to better capture cognitive activities that correlate with a human's trust in an autonomous system.

## 1 Introduction

Human-autonomy teams are expected to provide solutions in a wide range of applications, such as human directed search and rescue (Bashyal and Venayagamoorthy, 2008), hazard containment and mobilization (Nagatani et al., 2013), and space exploration (Fong et al., 2013). These teams consist of autonomous agents that coordinate their actions with the human partner to achieve a common goal (Mingyue Ma et al., 2018). Despite the advancements of current autonomous systems, it is the human's ability to engage their knowledge and experience that makes human-autonomy teams especially effective in tasks dominated by dynamic and uncertain conditions.

As autonomous systems become more sophisticated, the interaction between humans and these systems can be accurately described in terms of human-human teaming. Teammates must have a shared intent (Lyons et al., 2021), confidence in each other's capabilities, and similar focus of attention (Schaefer et al., 2017). Therefore, a broader investigation of human-autonomy interaction requires an examination of human factors such as workload, situational awareness (Musić and Hirche, 2017), and trust. Trust is a complex and multifaceted construct, yet one in which all humans are inherently familiar and capable of assessing qualitatively. It is commonly viewed as a latent variable that is not directly observable but must be inferred from other measures (Kohn et al., 2021). Within the context of a team-task, trust is established and maintained through the bi-directional interaction between one who evaluates the level of trust (a trustor) and one who impacts the level of trust (a trustee). Two key elements within the trusting interaction are the need for risk and the option for the trustor to be vulnerable. Trust is as a mental attitude or belief that evolves throughout the interaction, and is dependent on the interplay between analytic, analogical, and affective processes (Lee and See, 2004), especially emotional responses to violations or confirmations of expectations. The trustor continuously evaluates the trustee's desire and capability of acting benevolently to accomplish the team's task objective. As the task proceeds, each team member re-evaluates their trust in the other. Trust may change due to variation of task complexity, the transparency of the teammate, or a perception of their capability (Mayer and Davis, 1995). Therefore, trust is a dynamic process that is evaluated and updated constantly.

Humans teammates can establish trust both verbally, but more importantly non-verbally, through behaviors such as gestures and cues. However, when a human works with an autonomous system, the bi-directional interaction is effectively severed. Despite the universally accepted notion of trust, there is no universally agreed upon definition (Razin and Feigh, 2023). The human-autonomy literature frequently defines trust as “*the attitude that an agent will help achieve an individual's goals in a situation characterized by uncertainty and vulnerability”* (Lee and See, 2004). The factors affecting human trust in autonomy have been separated into three groups: human related, robot related, and environmental (Hancock et al., 2011). Human trust is initialized by their abilities such as competency and expertise, and personal characteristics such as attitudes or propensity to trust robots. Environmental factors establish the context and complexity of the team task. However, without the ability to interrogate their partners, the human's (trustor) subjective assessment toward the robot (trustee) is affected by the perception of competence as evidenced by the robot's behaviors, reliability, predictability, and the transparency of its actions.

Studies have shown that a human's miscomprehension of an autonomous system's state, decisions, or course of action can result in misuse or disuse of the agent, causing a reduction in team-task proficiency (Parasuraman and Riley, 1997). The correspondence between a human's trust in the agent and it's capabilities is known as trust calibration (Lee and Moray, 1994). Failures in human autonomy teaming can be the consequence of trust that exceeds, or is less than the system's capabilities. This degradation can be mitigated if trust between the human and autonomous agent (Chen and Barnes, 2014) is appropriately calibrated (de Visser et al., 2020). Furthermore, when the trust in autonomy is negatively impacted, it can be difficult for the human to regain it (Muir and Moray, 1996; Esterwood and Jr, 2023). Trust changes accordingly with the repeated interaction between the human and autonomous system (Schaefer et al., 2021; Tenhundfeld et al., 2022; Alhaji et al., 2025). Just as it is critical for the human to comprehend and predict the behaviors of an autonomous agent, it is equally critical for the autonomous agent to understand the cognitive state of the human in order to determine when, or potentially how, to communicate their own intentions or clarify their behavior (Dehkordi et al., 2021; Scheutz et al., 2022). Therefore, effective and efficient human-autonomy team-task performance can be significantly augmented if the autonomous agent has direct access to the internal cognitive state of the human both unobtrusively, and in real-time.

Neurophysiological correlates of human cognitive state have been studied using the electrical signals recorded directly from the surface of the scalp, known as an electroencephalogram (EEG) (Klimesch, 1999). EEG is a common, non-invasive measure of brain activity. Scalp voltages, on the order of 100μ*V*, correspond to average local neural activity. Typical EEG measures can be categorized into time domain features, frequency domain features, and functional connectivity metrics. Among these features, event-related potential (ERP) components are commonly used to understand subjects' neural responses toward specific task cues. Recent studies have explored how ERP components are correlated with trust (De Visser et al., 2018; Dong et al., 2015). The latter study highlighted that the two ERP components: Observational Error-related Negativity and Observational Error Positivity can combine the trust-relevant neural response with the subjects' assessment of autonomous performance. However, signal-power is this research area's most used frequency domain feature. Historical studies using EEG primarily investigate the magnitude and spatial distribution of signal-power within well established bandwidths: Delta (0.5–4 Hz, depth of sleep); Theta (4–8 Hz, working memory and cognitive fatigue); Alpha (8–13 Hz, relaxation and wakefulness); Beta (13–30 Hz, attention and motor execution); Gamma (>30 Hz, sensory integration) (Harmony, 2013). However, it is widely believed that cognition manifests through interactions between brain regions over a variety of spatial scales (Nikolaidis and Barbey, 2018). Synchronization of brain oscillations have been proposed as a key concept in neural processes underlying cognition (Gregoriou et al., 2015). Regions of the brain that exhibit statistical interactions in the absence of established neural pathways are known as “functionally connected regions.” The location of such brain regions and the statistical correlations between them establishes a topological network that can be succinctly described using elements of graph theory. Descriptive measures of network topology have been widely applied to EEG data. These analyses reveal non-random topological aspects, such as high clustering (Bullmore and Sporns, 2009), and metrics of dynamic functional connectivity may indicate changes in macroscopic neural activity patterns underlying critical aspects of cognition (Srivastava et al., 2022; Bales and Kong, 2022; Bales, 2023).

As there is no universally agreed upon definition of trust, there is also no universal way of measuring it. Much like a human-human interaction, it is assumed that the actual state of trust the human has in the autonomy is continuous. Typical methods of trust measurement are performed using surveys that are applied at various intervals (Yagoda and Gillan, 2012). These methods cannot adequately capture the continuous nature of trust. The human must remove themselves from the task and attend to the survey itself. Depending on the specific scenario, attending to a survey can range from inconvenient to absolutely detrimental to task performance, which in turn can have substantial impacts on trust. Similarly, surveys administered at the end of the task may only capture the human's net evaluation of trust over the length of the interaction. These methods lack the ability to measure trust both dynamically and unobtrusively. There has been substantial work attempting to indirectly measure trust continuously or semi-continuously through the use of physiological signals such as skin conductivity (Walker et al., 2019), heart rate (Waytz et al., 2014), and behaviors such as gaze (Hergeth et al., 2016), and interaction time (Akash et al., 2020). Nevertheless, behaviors are proxy measures and specific to the tasks for which they were measured. Trust is dependent on both cognitive and affective processes, and as a result, should be reflected in EEG. Cognitive state determined from EEG is specific to the human's physiological response and should generalize across a variety tasks more readily. In addition, it is possible that relevant changes in internal cognitive state precede changes in observed behaviors. Future autonomous system could use cognitive state estimates as soon as they become operationally relevant to communicate their intent to the human to support team task effectiveness (Lyons et al., 2021).

Existing work has investigated the neural correlates of trust by examining how average spectral band power relates to trust in various autonomous team settings (Wang et al., 2018; Oh et al., 2020, 2022; Akash et al., 2018). However, models utilizing only local neural activity do not address the possibility that communication between brain regions contributes to cognition, and that such contributions may occur even in the absence of changes in regional neural activity (Mišić and Sporns, 2016). *We hypothesize that a properly selected set of network-metric features derived from EEG measurements can predict human trust in an autonomous system with a higher accuracy than that of EEG signal-power features*. Several studies have explored variations in EEG functional connectivity as it relates to levels of trust in automated driving scenarios (Xu et al., 2022; Seet et al., 2022). However, these studies did not incorporate network-metrics into models for trust prediction.

To test our hypothesis, we conducted a human subject experiment to evoke changes in a human's trust in an autonomous system as they perform a team-task. Participants were instructed to self report their state of trust whenever they wish. We assume that when a participant chooses to self-report, a change in trust has occurred. The self report of trust allows the focus on the potential relevant cognitive processes that occur along with it. This method contrasts with existing studies in three ways: (1) The participant is allowed to guide us when to look for potential relevant changes in trust; (2) The participant is not disengaged from the task to fill out specific trust surveys at discrete times; (3) There is no aggregate evaluation of trust upon completion of the task. In addition, there have been appeals within the neuroscience community to treat the more peculiar aspects of “inter-subject diversity as signal, not as noise” (Viola, 2021). We compared the trust prediction accuracy of multivariate linear regression models using both EEG power and inter-channel functional connectivity features derived from a 62 channel EEG timeseries. In this study we do not hypothesize any mechanisms of EEG generation a-priori, and how specific EEG features change with trust will vary between individuals. Consequently, our study is within subject and the models generated are personalized.

To the best of our knowledge, this is one of the first studies to incorporate EEG inter-channel connectivity network based features in the prediction of dynamic changes of trust in a human-autonomy-team task.

## 2 Materials and methods

### 2.1 Experiment

#### 2.1.1 Ethics statement

This experiment was approved by the University of California, Davis Institutional Review Board. All participants were briefed on the experimental procedure and provided written informed consent prior to participating in the experiments.

#### 2.1.2 Participants

Ten students participated in this study: 6 males and 4 females, aged 21 to 31 years old, (*M* = 27, *SD* = 3). All participants were right handed and reported to have received 5 to 8 h of sleep the previous night, mean (*M* = 7.1, *SD* = 3.6). All completed the full experiment of 20 trials as described in Section 2.1.3. Each participant was briefed on the function of the instrumentation and testing procedure. Prior to the experiment, participants filled out a demographics survey which included questions regarding consumption of caffeine, prior experience with robotic or autonomous systems, and video gaming experience. All participants were compensated at a rate of $20 per hour.

#### 2.1.3 Task

**Figure 1:**
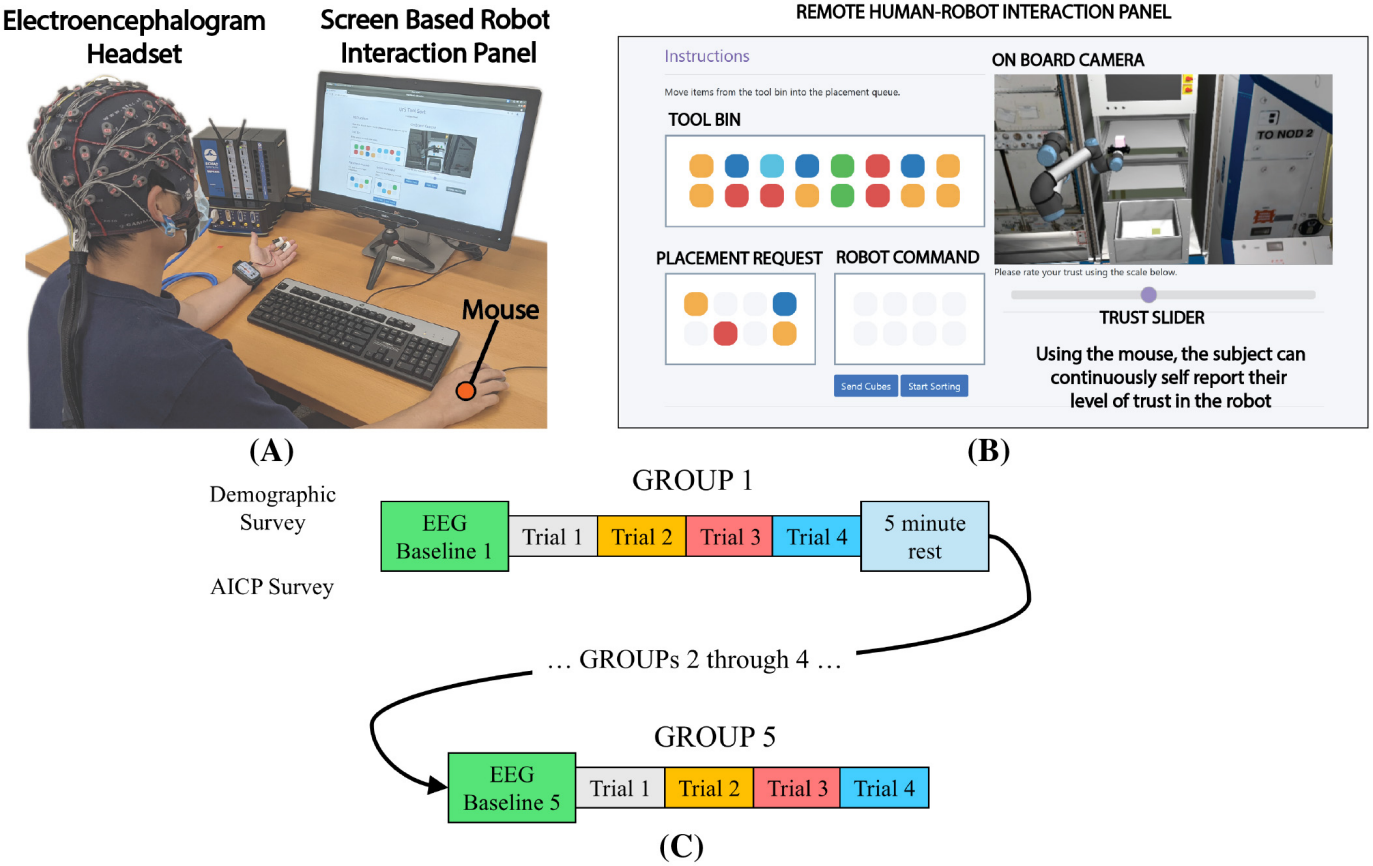
Details of the experiment **(A)** an image of the remote test panel used in this study. Each participant interacts with the system via the *Tool Bin, Placement Request*, and *Robot Command* panels. Cube placement is viewed through the *On Board Camera panel*. The participant signals changes in their level of trust using the *Trust Slider*. **(B)** Each participant was seated in front of a screen displaying the remote interaction panel. Brain activity was measured using an EEG device. In addition, gaze and mouse clicks were recorded. **(C)** Detail of the trials performed by each participant during the experiment. Each participant began with a baseline period, then moved through 5 GROUPs of 4 block placements. After each GROUP, participants were given an option to take a 5 min rest. Each new GROUP began with a new baseline recording. The total experiment lasted approximately one hour.

This experiment investigated how changing levels of human trust in autonomous systems are reflected in brain activity, specifically, scalp voltages measured with an EEG headset. We designed our screen based experiment using a ROS simulation shown in Figure 1A. Our custom interaction panel allowed the human participant to remotely oversee the placement of stowage onto an equipment rack by a UR5e robotic arm, ostensibly located on the International Space Station (ISS). Each participant was introduced to the fictional scenario whereby a critical maintenance task was to be performed by crew members onboard the ISS. The complete maintenance task was segregated to create a sense of interdependancey between individual elements of a broader team: (1) a procedure planning portion of the ground crew; (2) the human-robot team, comprised of the participant and the simulated robot; and (3) the onboard crew. The participant worked in collaboration with the robot to ensure the proper placement of stowage required for the onboard crew to perform the repair/maintenance task. Participants were instructed that the proper stowage placement was critical to the effective performance of the maintenance task.

Within the simulation, specific articles of stowage were represented as colored cubes. The cube colors were designed to be maximally distinguishable by participants with colorblindness. Participants were informed that the procedure planning team had developed a maintenance plan which required a sequence of cubes to be placed onto a rack. The sequence was communicated to the participant through the *Placement Request* panel. Using a mouse, the participant would sort the cubes from a *Tool Bin* by selecting the proper colored cube and placing it into the *Robot Command* queue. Once the queue was full, the participant clicked a button to start the robot placement sequence. The participant was informed that a planning algorithm decided how the cubes would be placed onto the rack. As a means to elicit changes in the participant's trust, the capability of proper stowage placement was modulated between two levels of placement accuracy: 90% and 30%. These levels were randomized and unknown to the participant. The two levels of accuracy ensured that each participant was exposed to the approximately the same number of correct and incorrect placements over the course of the experiment. As the robot performed the placement task, the participant was instructed to self-report their trust in the robot via a horizontal slider located directly under the *On Board Camera* panel. High trust was reported by positioning the slider to the right, whereas low trust was reported by positioning to the left. Trust values vary from 0 (no trust) to 1 (complete trust). Each participant was allowed to determine when, and by how much they decided to report their level of trust. Once the robot was finished placing the 4 cubes, the window was reset and a new sequence of cubes was displayed in the *Placement Request* panel.

It was necessary that our simulated robot was perceived by participants as an individual agent and a teammate, occupying a distinct role (Wynne and Lyons, 2018) that it performed uniquely, with capabilities that could be evaluated subjectively. In this way, the human and robot arm would be working interdependently toward the common goal. To enforce this, our simulation ensured that each placement trajectory experienced by participants throughout the experiment was unique. Each four block placement was planned independently. While placement accuracy was used to determine how many cubes would be placed in their proper positions, the order of cube placement and the trajectories of the robotic arm were calculated prior to the presentation of the cubes in the *Tool Bin*. Each of these trajectories were determined using a probabalistic roadmap motion planning algorithm, PRM* (Kavraki et al., 1996) via waypoints assigned above the start and finish position of each cube. No two trajectories were ever the same. As a result, participants had to rely on the robot based on both its performance and behaviors.

Participants self-report changes in trust based upon their perception of risk, task importance, and capability of the robot. Changes in self-reported trust are important in both direction and magnitude compared to previously reported levels. Previous works have similarly obtained dynamic reports of trust via periodic prompts to cue participants for a response of an increase, decrease, or no change in trust compared to the previous response (Desai, 2012; Desai et al., 2013). We assume self-report occurs when the participant has accumulated enough information about the state of the system and made the decision to report a change trust. If there is no self-report, we cannot impute a correspondence between a level of trust and EEG signal versus any other external or internal stimuli of brain activity. Self-reported trust is only a single dimensional measure of a complex concept. We do not claim that self-report trust levels are comparable between participants. The models constructed from our data are personalized, and the analysis that follows is within-subject. Despite limited research exploring the correlation between the trust slider and other established subjective trust reporting methods, it has been acknowledged (Kohn et al., 2021) that the likert or sliding scale is functionally similar to the trust item used in Lee and Moray's trust and self-confidence measure for measuring trust in automation (Lee and Moray, 1994).

#### 2.1.4 Apparatus

A detail of the experimental setup is shown in Figure 1A. Electrophysiological data were collected from each participant using a EEG recording suite manufactured by g.tec. The system was comprised of the g.HIamp amplifier and 62 channels of active electrodes mounted into a single flexible cap. Gaze position and pupil diameter were measured using a Tobii Nano Pro screen based gaze tracker. The gaze tracker captured pixel position of gaze, pupil diameter and blinks at a sample rate of 60Hz and is optimized for screen based experiments. In addition, mouse position and button clicks were recorded. Both the gaze and mouse data were used to observe the participants' interaction with the screen-based task. Additionally, gaze was used during baseline measurements that preceded each test block, and was crucial in identifying myoelectrical artifacts in the EEG signal due to blinking. All data were synchronized and recorded using Lab Streaming Layer at their native sample rates.

#### 2.1.5 Procedure

Prior to the experiment, all participants received guidance on executing the human-robot collaborative task using the screen based interface in Figure 1B. In addition, a training session was provided that introduced the background of the study including the scene, where the study took place, and the goal of the task. Furthermore, detailed instructions on how to evaluate trust according to the given task was highlighted to the participants. The instructions included two major constructs: (1) *We want you to report your trust as your attitude that the autonomous system will help you achieve your goals given the uncertainty and vulnerability associated with this task (and this task only)* (Lee and See, 2004) and (2) *Your trust may include aspects related to the performance of the autonomous system and also may include your feelings toward the autonomous system*. Thus, the participants were assumed to report their trust based on the same standard.

After receiving instructions, each participant was outfitted with the 62 channel EEG headset. The active electrodes were filled with conductive gel and electrode impedance was verified to be below 5kΩ using the g.tec data acquisition software. Next, the eye tracking device was calibrated for the particular participant. A complete experiment consisted of 5 GROUPs^1^ of 4 trials for a total of 20 placement trials as shown in Figure 1C. Prior to the first GROUP a baseline measure of EEG activity was recorded for approximately 4 min: 2 min with eyes open while fixating on a crosshair, and 2 min with eyes closed. The following four GROUPs starting with a similar baseline measure, but with eyes open/closed periods of 45 seconds each. After each GROUP, a 5 min rest period was provided. The total experiment lasted approximately one hour. A short video of a single trial is available (UC Davis CHPS Lab, 2022).

### 2.2 Methods

**Figure 2:**
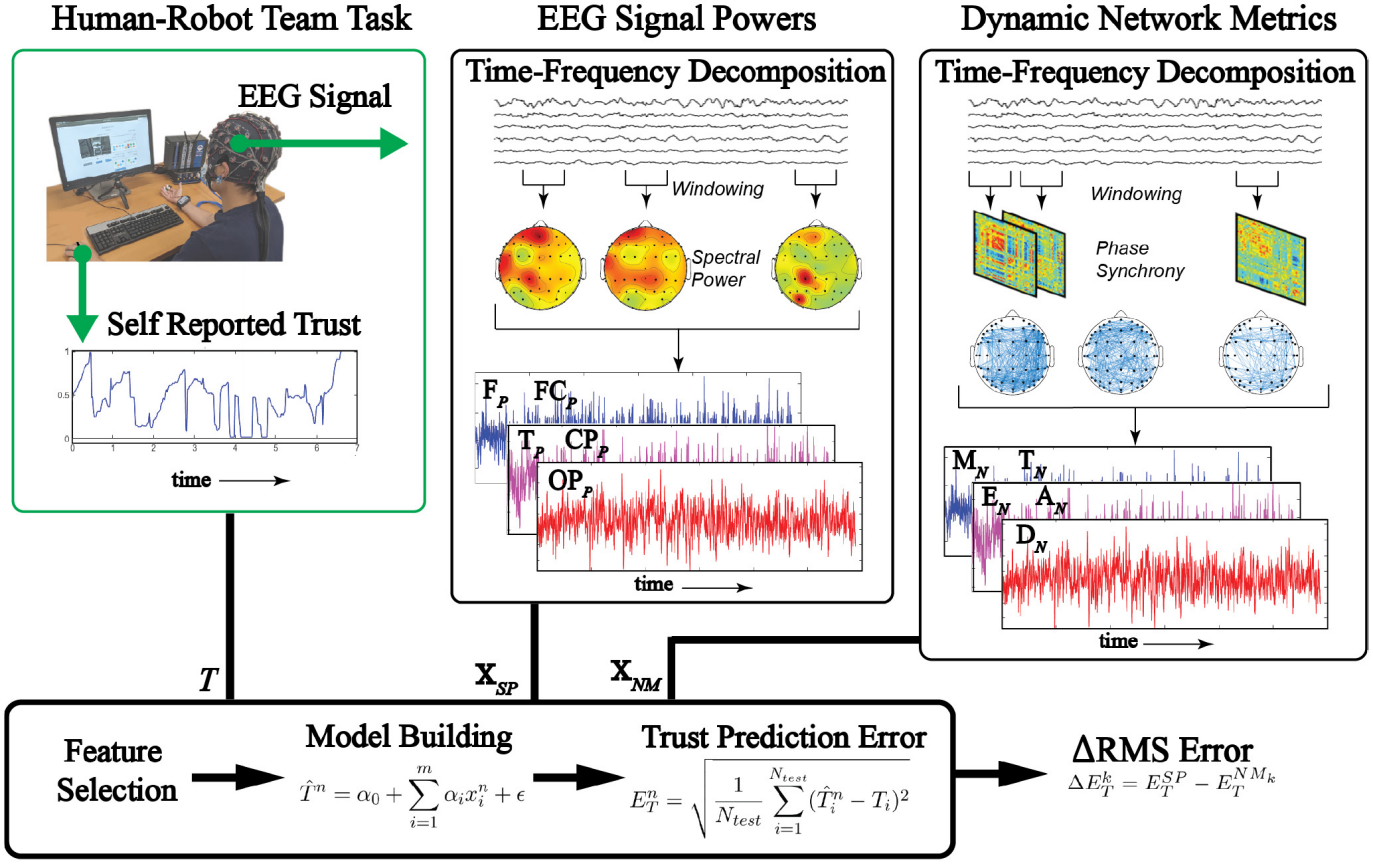
Data conditioning pipeline. EEG datawere recorded along with self reported trust. Features derived from network-metrics and single channel powers were selected as regressors. Linear models were constructed from the regressor sets and the results are compared.

We tested our hypothesis by comparing the trust prediction performance of linear models using regressors selected from two separate feature types derived from the EEG timeseries: (1) EEG signal-powers (SP); (2) EEG network-metrics (NM). In this section, we describe the methods used to generate our data and prepare it for analysis. The data analysis pipeline is shown in Figure 2.

#### 2.2.1 EEG features

Our EEG headset recorded 62 channels of scalp voltages at a sample rate of 512 Hz. Two electrode clips were attached to the right and left earlobes with an additional ground electrode required for active impedance control. All EEG signals were referenced to the right earlobe. The EEG data were filtered with a zero lag 4th-order bandpass filter (2–100Hz) with an additional notch filter (58–62Hz) for AC line noise. To reduce the impact of field spread, the EEG voltage data was transformed to current source density (μ*V*/*cm*^2^) based on a spherical spline surface Laplacian method (Perrin et al., 1989). We performed a time-frequency decomposition using Morlet wavelets (Sanei and Chambers, 2007) over a set Ω of 34 frequencies between 9.5 and 83 Hz. The time-frequency transform yielded a temporal sequence of complex values given by:


(1) $$\begin{array}{l} W\left(t,\omega_{i}\right)=A\left(t,\omega_{i}\right)e^{j\phi \left(t,\omega_{i}\right)} \end{array}$$

where ω_*i*_∈Ω. We extracted frequency dependent power *P* = *A*^2^ (μ*V*^2^/*cm*^4^) and phase ϕ (rad) directly from Equation 1 for each of the 62 channels in the EEG headset.

As we describe in Section 2.2.1.2, functional connectivity values are based on EEG inter-channel phase difference. For the sake of longer term fidelity of EEG signal phase, we chose not to use independent component analysis (ICA) to identify and remove myoelectrical artifacts (Makeig and Onton, 2011) due to blink events, which occurred infrequently. ICA can have indeterminate impacts on the phase content of a signal over the long time course (Thatcher et al., 2020). Additionally, the separate gaze data allowed us to specifically identify the occurrence of blinks and analyze their impact on the EEG signal. As a result, we ultimately chose to exclude frequencies below 9.5 Hz due to the excessive artifacts generated from blinking.

##### 2.2.1.1 EEG power

**Figure 3:**
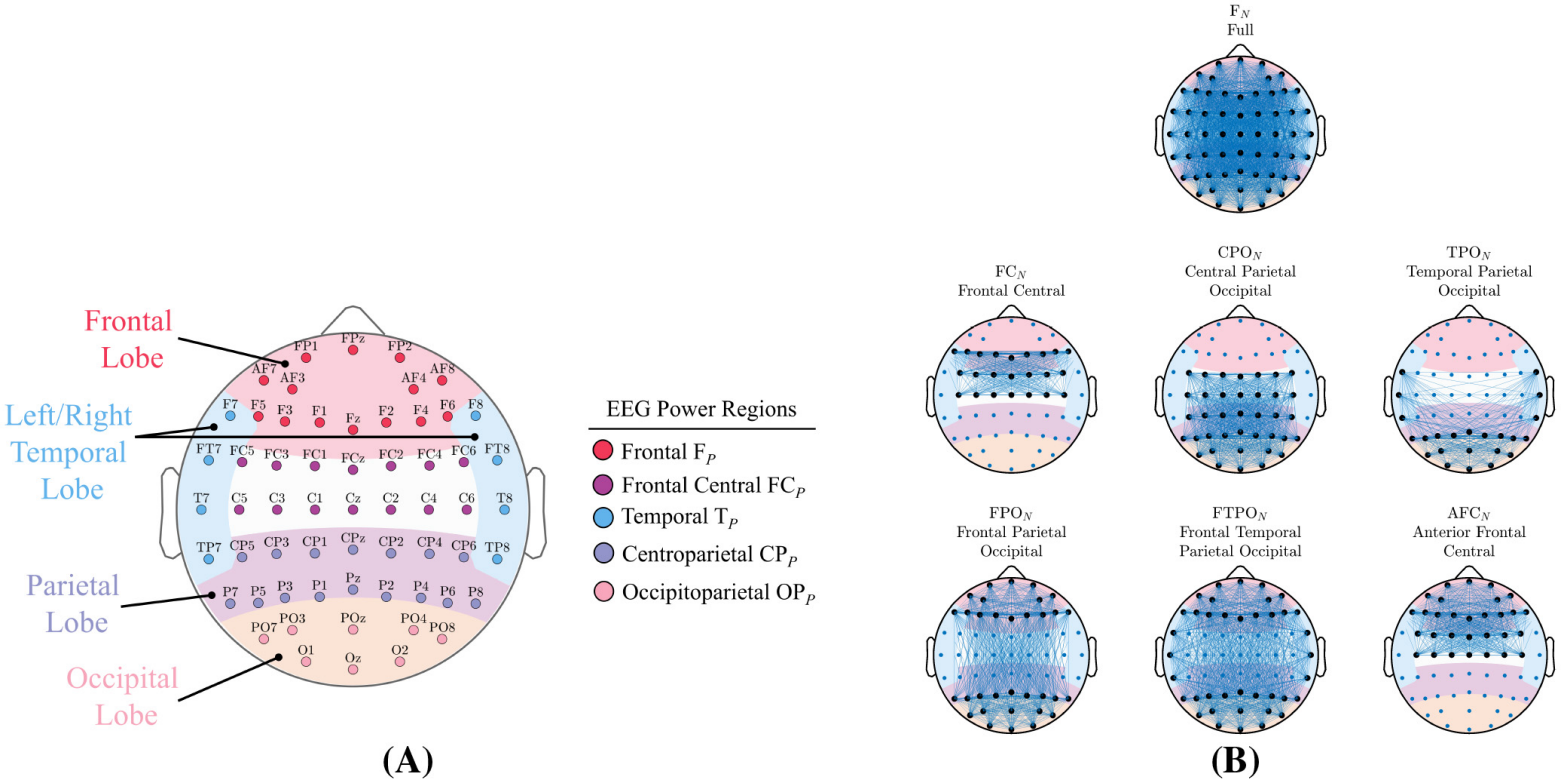
Signal-powers and network-metric feature types are generated from the regions shown. **(A)** The four primary brain lobes, and the five non overlapping EEG power-regions along with the 62 channels in our EEG device. **(B)** The seven region-networks analyzed in this study. Region nodes are shown with larger black markers while network edges are indicated by lines. Each headmap illustrates the complete number of edges that can exist in each region-network.

Figure 3A shows the 62 channels in our EEG headset and their locations over the four major lobes of the human brain: Frontal, Temporal (left and right), Parietal, and Occipital. We segregated the 62 EEG channels into five non-overlapping subsets located over these brain lobes. Details of each power-region is given in Table 1 including the names and abbreviations that will be used for the remainder of this paper. As described in Section 2.2.1, the channel powers were extracted directly from the time-frequency transform. Signal-power values for each region were determined as the average power over all channels in the region subset.

**Table 1:** Signal-power and network-metric feature details.

**Power region**	**Associated function**
Frontal F_*P*_	Behavior, emotions, judgment, planning, problem solving, concentration, and self awareness.
Frontal central FC_*P*_	Motor control and sensory perception.
Temporal T_*P*_	memory, hearing, sequencing, and organization.
Centroparietal CP_*P*_	Interpretation of language and words, interpretation and integration of sensory information, visual, and spatial perception.
Occipitoparietal OP_*P*_	Location integration and visual processing.
Network-metric	Description
Assortativity A_*N*_	A global correlation between nodes of high degree to nodes of low degree. Positive values indicate a network of mutually coupled high degree nodes, whereas negative values imply high degree nodes couple more with low degree nodes.
Density D_*N*_	The number of existing network edges as a fraction of the total number of possible edges.
Efficiency E_*N*_	A measure of the average inter-connectivity between different nodes in the network.
Modularity M_*N*_	A global measure of how the network forms separate, non-overlapping clusters.
Transitivity T_*N*_	A measure of the average tendency for nodes to group together into triangular clusters.

##### 2.2.1.2 Functional connectivity

In this investigation, we used phase synchrony (PS) as the measure of interaction between EEG electrode pairs. PS values describe the stability in signal phase difference between two electrodes over a given period of time. We calculated this quantity from the instantaneous phase difference between electrode pairs *l* and *m*


(2) $$\begin{array}{l} \Delta \phi^{lm}=\phi^{l}-\phi^{m} \end{array}$$

where ϕ is given by Equation 1. Two electrodes are considered synchronized over a time period Δ*t* = *t*_2_−*t*_1_ if $|\Delta \phi_{t_{2}}^{lm}-\Delta \phi_{t_{1}}^{lm}|<C$ for an angular threshold *C*. Rather than using a radian measure of Δϕ, we described synchronous behavior using the PS ψ_*lm*_, formally defined as follows:


(3) $$\psi_{lm}=\frac{1}{N_{T}}\left\|\sum_{i=1}^{N_{T}}e^{j\Delta \phi_{i}^{lm}}\right\|$$

where *N*_*T*_ is the number of discrete time points within a period Δ*t*. PS values vary from 0 to 1.

##### 2.2.1.3 Dynamic network-metrics

A network is defined as a binary undirected graph 𝒢 = (*V, E*) comprised of a set of nodes *V*∈(1, 2, …, *N*_*C*_) with *N*_*C*_ total nodes, and an edge set *E*⊆{(*x, y*)|(*x, y*)∈*V, x*≠*y*}. Each network node is mapped directly to an EEG electrode. For the remained of this paper, each electrode/node will be referred to as a channel.^2^ Hence, inter-channel connectivity refers to the PS values between electrode/node pairs. The network can be described via a symmetric square adjacency matrix $A\in {\mathbb{R}}^{N_{C}\times N_{C}}$. Elements of *A* are given as


(4) $$A_{lm}=\{\begin{array}{ll} 1 & \psi_{lm}>\delta_{PS}\\ 0 & otherwise \end{array}$$

where ψ_*lm*_ is the PS between channels *l* and *m* as described in Equation 3, and the parameter δ_*PS*_ encodes the maximum allowable change in phase difference. PS values between individual EEG channels were calculated over a sliding temporal window of Δ*t* = 0.4 s (Yoshinaga et al., 2020). PS values were thresholded at δ_*PS*_ = 0.89, yielding the adjacency matrix as shown by Equation 4. The presence of a network edge between two channels indicates the stability in their phase difference. The number of edges connected to a given node is known as the degree.

The distribution of edges and nodes can be summarized using global metrics that capture the structure, integration, and resilience of the entire network. Metrics of assortativity (A_*N*_), density (D_*N*_), transitivity (T_*N*_), efficiency (T_*N*_), and modularity (M_*N*_) were calculated using the Brain Connectivity Toolbox (Rubinov and Sporns, 2010) in MATLAB. Properties of each individual metric are outlined in Table 1. Values of network-metrics do not have unique one-to-one associations with specific distributions of network edges. Rather, they capture average properties of the entire network. The resulting time series represented the dynamic changes in network properties throughout the human-robot team task.

##### 2.2.1.4 Region-networks

The 62 channel EEG system provided an opportunity to explore complex interconnections between distant brain regions. In addition to the full 62 channel ensemble, we analyzed six region-networks as shown in Figure 3B which lists the names and abbreviations that will used for the remainder of this paper. Region-networks were comprised of channel subsets located over particular brain lobes that are known to functionally interact. The region-networks shown in Figure 3B are complete graphs and represent the total number of possible edges that could exist in that region-network.

##### 2.2.1.5 Feature counts

For a direct comparison of model results between feature types, the number of features generated from the signal-powers and network-metrics were identical. The SP features were frequency dependent powers over the five separate EEG regions. Likewise, the NM features were the five frequency dependent metrics. Both sets of EEG derived measures were defined for each of the 34 frequencies in Ω for a total of 170 features. The signal-power regressor set will be denoted as **X**_*SP*_, and network-metric regressor set as ${\text{X}}_{NM}^{k}$, where *k* is one of the seven region-networks listed in Figure 3B.

#### 2.2.2.Linear model

In this study, we assumed that the state of trust was based upon the participants' perception of risk, task importance, and capability of the robot to properly perform the placement task. We additionally assume that both trust and EEG features were continuous. Therefore, our predictive models assume a consistent correlation between the *m* independent variables, EEG features *x*_*i*_, and the dependent variable, trust *T*, of the form shown in Equation 5. Regressors were selected from the complete feature sets for each feature type: *x*_*i*_∈**X**_*SP*_ for the SP model, and $x_{i}\in {\text{X}}_{NM}^{k}$ for the seven region-networks. We constructed a series of multivariate linear models from each feature set as follows:


(5) $$\begin{array}{l} {\hat{T}}^{n}=\alpha_{0}+\overset{m}{\underset{i=1}{\sum }}\alpha_{i}x_{i}^{n}+\epsilon \end{array}$$

where ${\hat{T}}^{n}$ is the trust estimate using regressors from one of the eight feature types, *m* is the total number of regressors in the model, *a*_0_ is a bias term, α_*i*_ are the coefficients of each regressor $x_{i}^{k}$, and ϵ is a random noise term. We additionally assumed that interactions between features were negligible. Personalized linear models were generated for each participant using regressors selected from the SP feature set **X**_*SP*_ and seven NM feature sets ${\text{X}}_{NM}^{k}$ for a total of eight regressor sets for each participant.

##### 2.2.2.1 Feature selection

Selection of the features $x_{i}^{k}$ in Equation 5 was accomplished using a greedy feed-forward search approach implemented using the MATLAB function sequentialfs.m with root mean squared (RMS) estimation error as the loss function (MATLAB, 2022). The algorithm begins with a constant term *a*_0_ and sequentially adds regressors until the relative reduction in RMS error met a selected threshold (Kuhn and Johnson, 2013). Feature selection was performed for each of the eight feature sets, **X**_*SP*_ and ${\text{X}}_{NM}^{k}$, as described in Section 2.2.1.5. The total number of regressors used for all models in this study wa *m* = 30.

#### 2.2.3 Participant trust levels

A major assumption in this study was that elicited changes in trust occurred over large timescales such they may be captured by examining average EEG measures at discrete points in time. Our participants self reported their trust from 0 (no trust) to 1 (complete trust), *T*_*m*_∈[0, 1]. We use the temporal characteristics of *T*_*m*_ to define a discrete set of time points *t*_*r*_, from which to build our models. Each participant's self reported trust over the entire experiment was collected into a single ensemble.

#### 2.2.4 Model generation

Our method of building and testing the performance of the linear models was based on N-fold cross validation. Sample points for each participant over an entire experiment were collecting into a single ensemble. All sampled points were randomly assigned to one of ten equally sized bins: *S* = {*s*_1_, *s*_2_, *s*_3_, …, *s*_10_}. A 10% holdout, *S*_*test*_, of data was set aside testing while the remaining 90% *S*_*train*_ was used for model building. If we define the set *B* = {*b*_1_, *b*_2_, *b*_3_, …, *b*_*N*_*B*__} where $b_{i}=\left\{S_{train}^{i},S_{test}^{i}\right\}$, there are NB=mm10 unique, non-overlapping sets in *B*. We built and evaluated linear models using 1,000 randomly selected train/test sets *b*_*i*_∈*B* using the MATLAB function fitlm.m. For each set *b*_*i*_, we constructed *n* = 8 models from the ranked regressor sets: one model for signal-power, and seven models for the region-networks. Prior to model construction, all trust and EEG data were temporally aligned to ensure common time stamps between signals of different sampling rates. During the model building phase, normality of the residuals was verified using a Shapiro-Wilk test.

#### 2.2.5 Statistical analysis

The regressor types, whether SP or NM, are derived from EEG signals that measure neural activity arising from the same stimulus. Consequently, we assumed that model performance for all regressor types represents an ensemble of possible predictive performance using EEG for each individual participant.

Trust prediction accuracy was evaluated using the root mean squared error between the self reported and model predicted values of trust using the test set $S_{test}^{i}$ that was unseen during the model building phase. The root mean squared trust prediction error using models built from the *n*^*th*^ regressor set will be denoted as $E_{T}^{n}$ and is given by


(6) $$\begin{array}{l} E_{T}^{n}=\sqrt{\frac{1}{N_{test}}\overset{N_{test}}{\underset{i=1}{\sum }}{\left({\hat{T}}_{i}^{n}-T_{i}\right)}^{2}} \end{array}$$

where ${\hat{T}}_{i}^{n}$ is the trust estimate, *T*_*i*_ is the true self reported trust, and *N*_*test*_ is the number of points in the test set. The $E_{T}^{n}$ is the standard deviation of the trust prediction error. Given the range of *T*_*m*_ = [0, 1], a value of $E_{T}^{n}$ = 0.25 for example, would indicate that estimated trust could differ from the actual trust by 25%. Additionally, we compared adjusted *R*^2^ to determine how well regressor types capture the variability in self reported trust.

Despite the presence of normally distributed residuals during the training phase, there was a high incidence of skewed distributions of $E_{T}^{n}$ in the prediction results. This trend was also present for the adjusted *R*^2^. Consequently, we compared the medians of $E_{T}^{n}$ and adjusted *R*^2^ between SP and NM feature types using a non-parametric Wilcoxon rank-sum test for equal medians with a significance level of α = 0.05.

## 3 Results

This study proposed that personalized predictive models of a human's trust in an autonomous system would perform better using inter-channel EEG network-metrics over traditional EEG signal-powers. In this section we present the results as they pertain to the two feature types. Personalized models were developed for each of the 10 participants in our cohort. We compared the trust prediction errors for the SP model type against those of the seven region-network model types for a total of 70 comparisons.

### 3.1 Trust response

**Figure 4:**
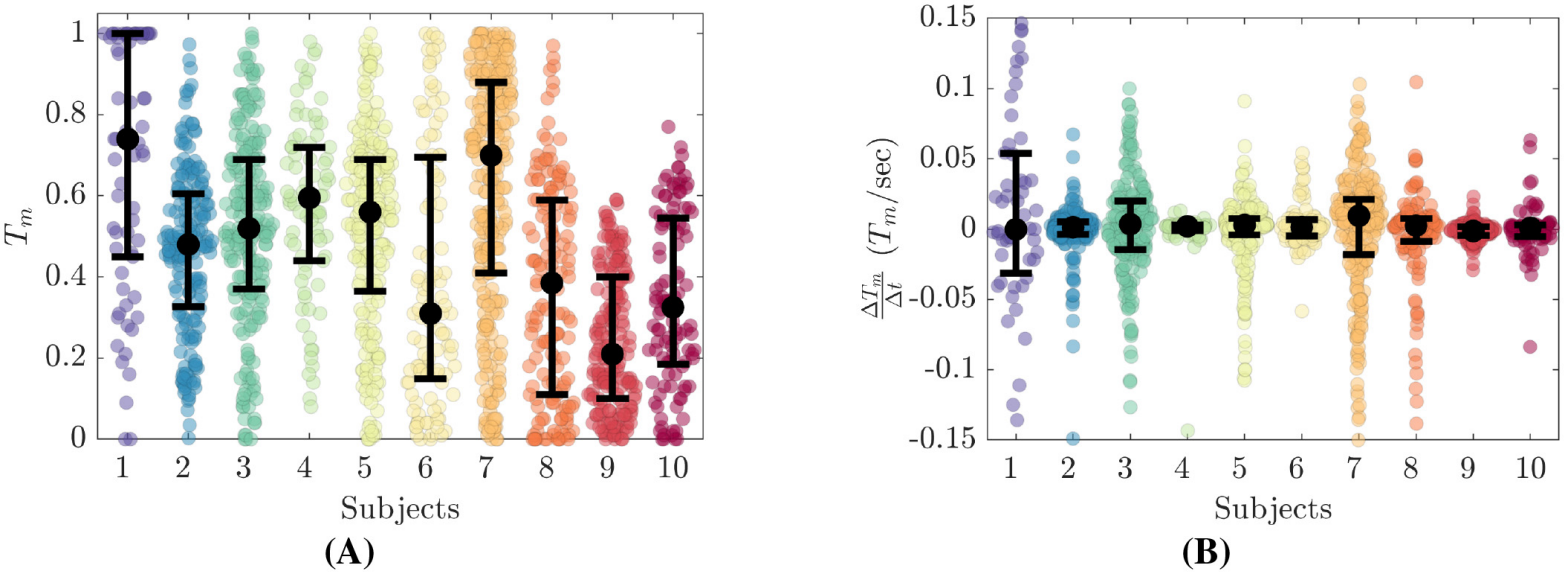
Trust reporting details for all participants. **(A)** Distributions of self reported trust, *T*_*m*_. **(B)** Distributions of trust derivative, $\frac{\Delta T_{m}}{\Delta t}$.

The characteristics of each participant's trust reports are highlighted in Figure 4. The distribution of trust reports is given in Figure 4A with units of *T*_*m*_∈[0, 1]. Figure 4B shows the numeric derivative of trust ($\frac{\Delta T_{m}}{\Delta t}$), which we defined as the change in *T*_*m*_ divided by change in report time. Black dots represent the median values and the interquartile range (IQR) is denoted by the lower and upper bars. These distributions highlight the differences between each participant's trust reporting preferences. The number of trust reports for each of the participants is given in Table 2. The number of reports range from 66 to 281 (*M* = 158, *SD* = 69) over the course of each experiment.

**Table 2:** Detail of trust reports.

**Variable**	**Statistic**	**Subject**									
		**1**	**2**	**3**	**4**	**5**	**6**	**7**	**8**	**9**	**10**
*T*_*m*_	Count	66	192	219	78	208	104	281	150	178	108
*T*_*m*_	Median	0.74	0.48	0.52	0.60	0.56	0.31	0.70	0.39	0.21	0.33
$\frac{\Delta T}{\Delta t}$	IQR	0.085	0.009	0.034	0.004	0.012	0.011	0.040	0.016	0.006	0.008

From Figure 4A, we see that each participant's trust reports cluster about their own personal medians. Eight of the ten participants reported *T*_*m*_ over the full span of [0, 1], while Subjects 9 and 10 did not report *T*_*m*_ above 0.6 and 0.8 respectively. Subjects 1 and 7 report much higher *T*_*m*_ while Subjects 6, 8 and 9 report much lower. Subjects 2 through 5 have median *T*_*m*_ between 0.48 to 0.60. The highest median *T*_*m*_ was reported by Subject 1 at 0.74. All Subjects reported values of *T*_*m*_ = 0, while only Subjects 1 through 8 reported values up to *T*_*m*_ = 1. Subject 9 and 10 did not report *T*_*m*_ greater than 0.6 and 0.78 respectively.

Figure 4B shows that all participants had relatively symmetric distributions of $\frac{\Delta T_{m}}{\Delta t}$ centered about 0. The IQR values of $\frac{\Delta T_{m}}{\Delta t}$ vary from minimum values of 0.004 and 0.006 for Subjects 4 and 9 respectively, to maximum values of 0.085 and 0.040 for Subjects 1 and 7 respectively.

### 3.2 Model performance

**Figure 5:**
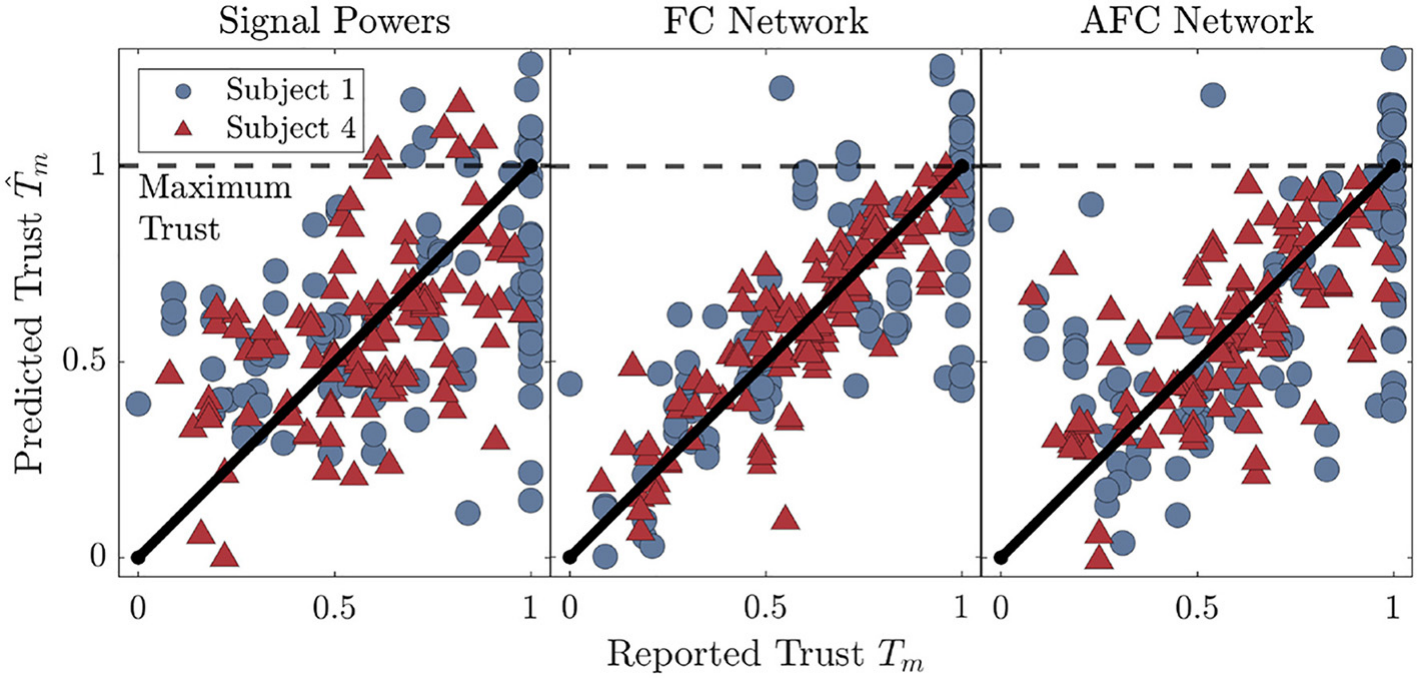
Trust prediction results for two of the participants in our cohort. The results are shown for the SP models, as well as the highest (FC_*N*_), and lowest (AFC_*N*_) performing region-networks. The maximum possible trust report at *T*_*m*_ = 1 is marked with the horizontal dotted line while the diagonal line represents perfect prediction.

To visualize the trust prediction behavior for the different feature types, Figure 5 details the results for two of the participants in our cohort. Prediction results from SP and NM regressor based model are shown for both the highest (FC_*N*_) and lowest performing (AFC_*N*_) region-networks. The diagonal black line in each plot represents perfect prediction. Vertical deviations from the line are trust prediction errors. These plots highlight some of the individual differences in trust prediction accuracy that are not necessarily captured in the $E_{T}^{n}$ values. Most notably, Subject 1 has a large number of reports close to *T*_*m*_ = 1, and models using both feature types performed poorly for high values of trust. In contrast, models for Subject 4 predict trust well over the entire trust range.

#### 3.2.1 Trust prediction performance

**Figure 6:**
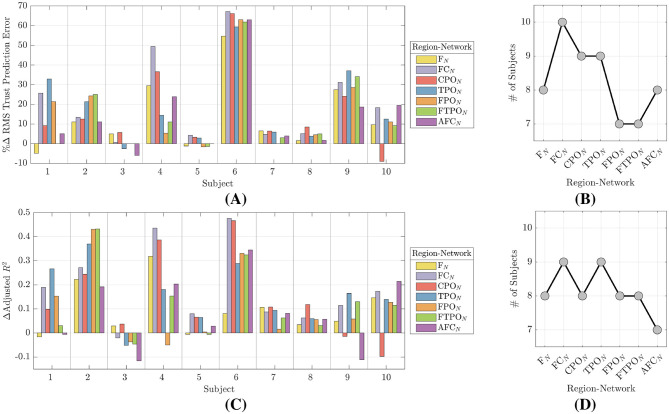
Trust prediction error results. **(A)** Percent changes in RMS trust prediction error between SP and NM model types. Data is shown for each participant across all region-networks. Positive values indicate that NM features more accurately predict trust. **(B)** Number of participants with reduction in trust prediction error using NM features by region-network. **(C)** $\Delta R_{k}^{2}=R_{k_{N}M}^{2}-R_{SP}^{2}$. Data is shown for each participant across all region-networks. Positive values indicate that NM features more adequately describe the variability in self reported trust. **(D)** Number of participants with increase in adjusted *R*^2^ using NM features by region-network.

Our study investigates the difference in trust prediction accuracy between linear models using SP and NM regressors. We evaluated model predictive power by comparing the RMS trust prediction errors as defined by Equation 6. Data in Table 3 is given as the difference in median $E_{T}^{n}$ between the SP model and seven NM models for the region-networks: $\Delta E_{T}^{k}=E_{T}^{SP}-E_{T}^{NM_{k}}$. Positive values of $\Delta E_{T}^{k}$ indicate a smaller trust prediction errors for NM based models and consequently improved trust prediction accuracy. The resulting *p* values are given for comparisons that failed the statistical test. Figure 6 illustrates our main findings. The percent change in trust prediction error, defined as $\frac{E_{T}^{SP}-E_{T}^{NM_{k}}}{E_{T}^{SP}}$, is given in Figure 6A. Figure 6B shows how many participants' models yielded improved trust prediction accuracy when using NM features. Counts are given for each of the seven NM region-networks.

**Table 3:** ΔRMS trust prediction errors: $\Delta E_{T}^{k}=E_{T}^{SP}-E_{T}^{NM_{k}}$.

**Region-network** ^a^	**Subject**									
	**1**	**2**	**3**	**4**	**5**	**6**	**7**	**8**	**9**	**10**
F_*N*_	–0.014	0.023	0.010	0.063	–0.003	0.425	0.018	0.004	0.052	0.018
FC_*N*_^b^	0.071	0.028	0.001	0.105	0.010	0.523	0.013	0.011	0.059	0.034
CPO_*N*_^b^	0.025	0.026	0.012	0.078	0.007	0.515	0.018	0.019	0.045	–0.017
TPO_*N*_^b^	0.091	0.045	-0.005	0.031	0.006	0.462	0.016	0.009	0.070	0.023
FPO_*N*_	0.059	0.051	*p=0.91*	0.011	–0.004	0.491	*p=0.32*	0.010	0.054	0.020
FTPO_*N*_	*p=0.92 *	0.053	*p=0.07*	0.023	–0.003	0.481	0.008	0.011	0.064	0.017
AFC_*N*_	0.014	0.023	–0.013	0.051	*p=0.46*	0.490	0.011	0.004	0.035	0.036

^a^ Unless otherwise stated, all *p* < 0.001

^b^These are the highest performing region-networks as discussed in Section 3.0.3.

Participants with lowest improvement were Subject 3 at 1%–5%, Subject 5 at 3%–4%, Subject 7 at 3%–7%, and finally Subject 8 at 2%–8%. The greatest improvement was Subject 6 at 55%–67% followed by Subject 9 at 19%–35%. Of the seven region-networks, only F_*N*_ showed a decrease in RMS trust prediction error for all ten participants. This was followed by CPO_*N*_ and TPO_*N*_, which showed decreases in nine participants; FN_*N*_ and AFC_*N*_ in eight participants; and FPO_*N*_ and FTPO_*N*_ in seven participants.

#### 3.2.2 Adjusted *R*^2^

In addition to trust prediction error, we calculated the coefficient of determination (adjusted *R*^2^) (Kuhn and Johnson, 2013) to compare the proportion of variance in self reported trust that was captured by models using SP and NM regressors. These results are also shown in Figure 6. Figure 6C gives the difference in median adjusted *R*^2^ between the SP model and seven NM models for the region-networks: $\Delta R_{k}^{2}=R_{k_{N}M}^{2}-R_{SP}^{2}$. A positive value of $\Delta R_{k}^{2}$ indicates that NM based regressors better capture variability in the self reported trust. All median differences were statistically significant with *p* < 0.001. Figure 6D shows how many participant's models produced higher adjusted *R*^2^ values when using NM features. Counts are given for each of the seven NM regions.

Participants that showed positive increases in adjusted *R*^2^ for all seven of the region-networks were Subjects 2, 6, 7, and 8. Subjects 4 and 10 had a single region that yielded a reduction in adjusted *R*^2^ while Subjects 1, 5, and 9 had two regions that produced a reduction. Subject 3 had the poorest performance with five of the seven region-networks showing a reduction in adjusted *R*^2^. The participants with the greatest improvements were Subjects 2, 4, and 6, with Δ*R*^2^ of 0.2 to 0.45. The participants with the smallest improvements were Subjects 5, 7, and 8 with 0.05 to 0.10. Subject 3 had only two regions that showed an increase in adjusted *R*^2^ at 0.05.

Of the seven region-networks, FC_*N*_ and TPO_*N*_ showed improvements in nine participants. The AFC_*N*_ region-network showed improvements in seven participants, while the remaining region-networks showed improvements in eight participants.

#### 3.2.3 Patterns in performance and trust reporting behavior

Patterns in trust reporting behavior and model performance are summarized in Table 4. The first row orders participants based upon their average ΔRMS trust prediction errors across all region-networks from lowest to highest. Similarly, the second row orders participants by their average Δ*R*^2^ across all region-networks from lowest to highest. Finally the third row orders the participants by their $\frac{\Delta T_{m}}{\Delta t}$ IQR from highest to lowest. Subjects 3, 5, 7, and 8 are in the bottom half of ranked performance measures. In addition, Subjects 3, 5, 7, and 8 are in the top half of ranked $\frac{\Delta T_{m}}{\Delta t}$. Participants who reported trust more frequently and with larger magnitudes had the lowest performing models using EEG-derived features of both types.

**Table 4:** Subject rankings of model performance and behavior.

	**Subject ranking**									
$\%\Delta E_{T}^{k}$ Low → High	**3**	**5**	**8**	**7**	10	2	1	4	9	6
ΔAdjusted*R*^2^ Low → High	**3**	**5**	9	**8**	**7**	1	10	4	2	6
$\frac{\Delta T_{m}}{\Delta t}$ IQR High → Low	1	**7**	**3**	**8**	**5**	6	2	10	9	4

Bold values indicate subjects with the lowest model performance and highest trust derivative.

### 3.3 Ranking the feature types

**Figure 7:**
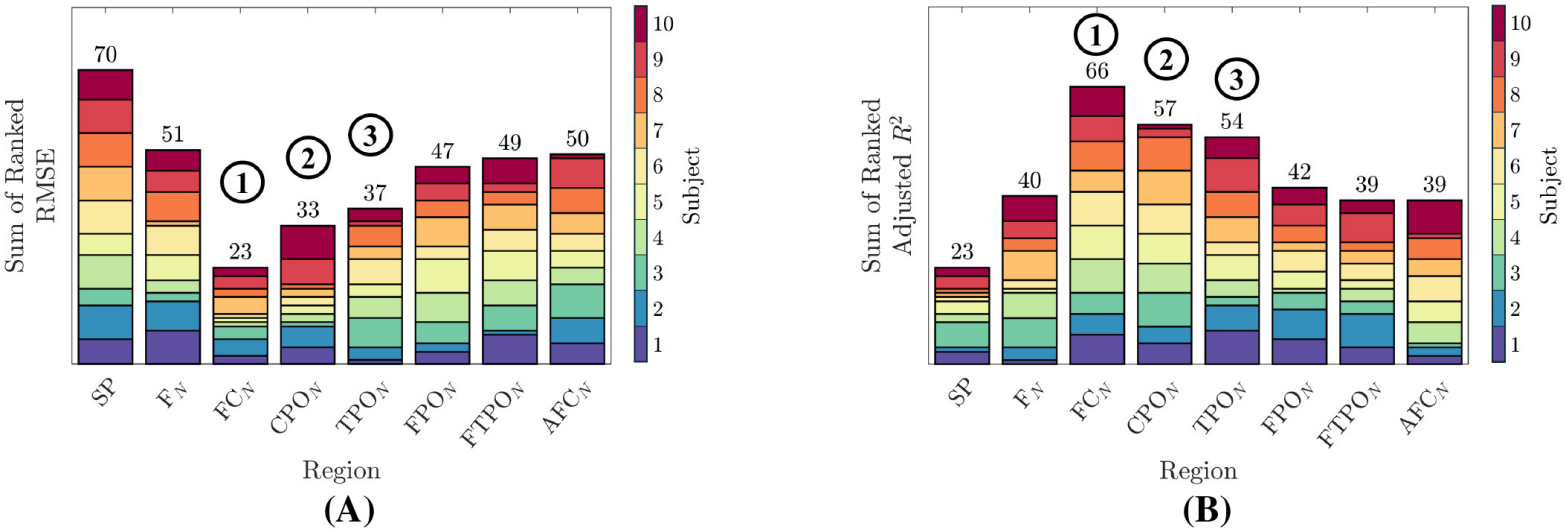
Ranking the performance of each regressor type over all participants. Each participant's $E_{T}^{n}$ and adjusted *R*^2^ values are ranked according the performance of their 8 model types. Higher ranks for adjusted *R*^2^ indicate better model performance, while lower ranks for $E_{T}^{n}$ indicate better model performance. **(A)** $E_{T}^{n}$ ranked for each participant from lowest to highest. The top three performing regions with the lowest ranked $E_{T}^{n}$ are highlighted. **(B)** Adjusted *R*^2^ ranked for all participants from lowest to highest. The top three performing regions with the highest ranked Adjusted *R*^2^ are highlighted.

In this study we generated personalized predictive models of trust and quantitatively compared the results within-subject. Regardless of the absolute values of $E_{T}^{n}$ and adjusted $R_{n}^{2}$, we assigned a numeric rank to those values within each participant's ensemble, from 1 to 8 for lowest to highest. Ranks for each of the regressor types were summed across all participants. The summed ranks are shown in Figure 7 as a stacked bar plots. Summed ranks for $E_{T}^{n}$ are given in Figure 7A, where lower values for a given regressor type indicate lower individual $E_{T}^{n}$, and therefore, better trust prediction accuracy. Summed ranks of $R_{n}^{2}$ are given in Figure 7B. Higher values of ranked $R_{n}^{2}$ show higher values of $R_{n}^{2}$ per participant, and consequently, models that better capture the variance in *T*_*m*_.

The top three ranked feature types in terms of lower $E_{T}^{n}$ and higher adjusted $R_{n}^{2}$ are network-metrics generated from the FC_*N*_, CPO_*N*_, and TPO_*N*_ region-networks.

### 3.4 Feature importance

**Figure 8:**
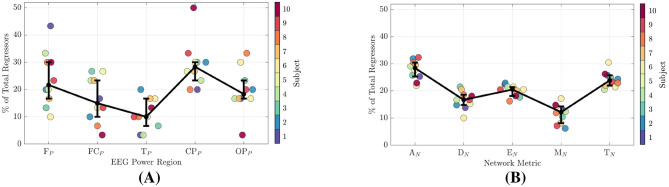
Signal-power and network-metric feature importance for all participants. **(A)** EEG signal-power feature importance. **(B)** Network-metric feature importance.

The feature selection algorithm described in Section Section 2.2.2.1 added regressors to the model only if they increased trust prediction accuracy. In this section, we report the number of occurrences of each feature type within the selected set of 30 ranked regressors. Features were separated into the two main types described in Table 1. Figure 8 illustrates the distribution of features types for all participants regardless of frequency. Signal-power feature counts are shown in Figure 8A. Network-metric feature counts are given in Figure 8B. Metrics capture the same qualities of a network regardless of the number of nodes/channels. Therefore, feature counts for all region-networks were collected together. Median values of each feature type are given by black dots and the interquartile range is denoted by the lower and upper bars.

**Figure 9:**
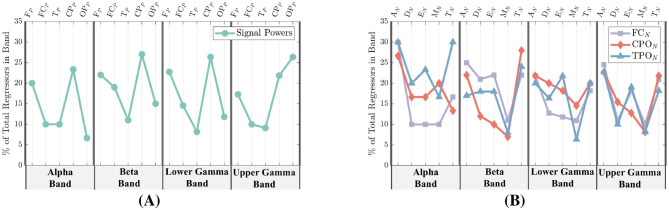
Signal-power and network-metric feature importance for all participants separated into the frequency bands of Alpha (9.5–13Hz), Beta (13–30Hz), Lower Gamma (30–48Hz), and Upper Gamma (48–83Hz). **(A)** EEG power feature importance. The most common signal-power regions selected among all participants are the F_*P*_ and CP_*P*_ regions. **(B)** Network-metric feature importance. The most common network-metrics selected among all participants are the A_*N*_ and T_*N*_.

In Figure 9, the feature counts are further broken down into four separate frequency bands: Alpha (9.5–13Hz), Beta (13–30Hz), Lower Gamma (30–48Hz), and Upper Gamma (48–83Hz). Frequency bands are labeled at the bottom of each figure, while the specific features are given at the top. Signal-power feature counts are shown in Figure 9A, and network-metric feature counts are given in Figure 9B. Unlike Figure 9B, the network-metric feature counts are further separated into the three top ranked region-networks.

#### 3.4.1 EEG signal-powers

From Figure 8A, the power region with the highest number of regressors is the CP_*P*_ at 28%, followed by the F_*P*_ at 22%, OP_*P*_ at 18%, and then the FC_*P*_ and T_*P*_ regions at 15% and 10%, respectively. As shown in Figure 9A, when power features are separated by frequency band, the CP_*P*_ and F_*P*_ regions remain the top contributors, accounting for 20%–26% of regressors across the Alpha through Lower Gamma bands. In the Upper Gamma band, however, the most important regressors shift to the OP_*P*_ region, followed by the CP_*P*_.

#### 3.4.2 Network-metrics

In Figure 8A, the network-metric types with the highest count are A_*N*_ at 28% followed by T_*N*_ at 24% and E_*N*_ at 20%. Modularity (M_*N*_) and Density (D_*N*_) were selected at 16% and 12% respectively. When additionally separating the metrics by frequency among the highest performing region-networks, A_*N*_ and T_*N*_ are within the top three selected metrics for all regions and bands with the exception of CPO_*N*_ in the Alpha band as seen in Figure 8B. Similarly, Efficiency is within the top three for all but the FC_*N*_ in Lower Gamma, and the CPO_*N*_ in Beta and Upper Gamma.

## 4 Discussion

This study investigated methods for predicting dynamic changes in human trust in autonomy using EEG-based metrics derived from network science, with the goal of enhancing human-autonomy interaction in team-task scenarios. Previous work in the fields of psychology and neuroscience has indicated that cognition arises through complex interactions between neural regions of the brain, and that these interactions can be reflected in inter-channel synchrony of EEG timeseries. We posited that features derived from dynamic inter-channel EEG networks contain information that would predict a human's trust in an autonomous system better than traditional measures of neural activity based on local EEG powers. We developed linear regression models to predict participants' trust levels as they engaged in a joint sorting task with a simulated robot. Unlike previous work, our analysis focused not on feature magnitudes, but on their correlation with changes in trust. In this section we discuss our findings.

We compared the performance between trust models using SP features, and NM features derived from seven separate region-networks. Both RMS trust prediction error and adjusted *R*^2^ were used to evaluate model performance. Unlike other studies, participants were allowed to report changes in trust freely, leading to individual assessments of the autonomous system that varied across participants. Models were personalized, based on each individual's unique experience with the system. Our findings indicate that each participant's trust reporting behavior ultimately limited the predictive performance of EEG-derived features. Nevertheless, network-metrics generated from the FC_*N*_, CPO_*N*_, and TPO_*N*_ outperformed signal-power models regardless of variations in trust patterns. Furthermore, certain feature types were consistently selected across all participants.

Signal-power features selected as having the greatest predictive performance were located in the F_*P*_ and CP_*P*_ regions across the Alpha through lower Gamma frequency bands, and the OP_*P*_ region in the upper Gamma band. Our results are congruent with other studies that have identified neural correlates of trust in similar brain regions. For example, Alpha and Beta band power in the frontal lobe (Oh et al., 2022), and a broad wave band from the frontal and occipital lobes (Wang et al., 2018). Another study found a reduction in Alpha power over parietal electrodes predicted decisions to trust (Blais et al., 2019). We will not interpret the meaning of these signal-power feature types as this has been explored in many other studies regarding trust (Hopko and Mehta, 2021).

Network-metrics selected as having the greatest predictive performance were A_*N*_, T_*N*_, and to a lesser extent E_*N*_, across all frequency bands. Several studies have explored changes in metric values with high and low trust in autonomous driving scenarios. For example, Seet et al. (2022) found that the Alpha band clustering coefficient (similar to transitivity) in the right frontal lobe was greater for more trustworthy system behavior. Similarly, Xu et al. (2022) found a decrease local efficiency and small-worldness (impacted by global efficiency) in the Beta band during less trustworthy system behavior. However, there is little research that investigates the potential implications of the specific network-metric values as they pertain to trust prediction. Outside of the trust literature, the dense functional inter-connectivity in the frontal lobe, and the dynamic reconfiguration of these connections has been shown to predict performance in a working memory task (Braun et al., 2015). In other studies, network-metrics have been utilized to predict general cognitive ability (Molloy et al., 2025; Popp et al., 2024).

**Figure 10:**
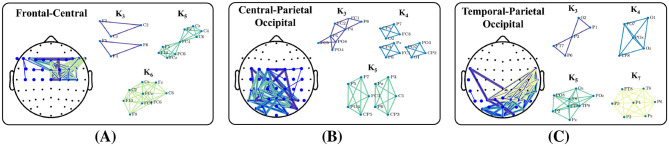
Examples of the networks that generated the metrics selected. Each headmap shows the inter-channel connections that generated the A_*N*_, T_*N*_, E_*N*_, and D_*N*_ values shown. **(A)** FC_*N*_ network and cliques **(B)** CPO_*N*_ network and cliques **(C)** TPO_*N*_ network and cliques.

Interpretation of the network features must begin with the definition of network edges (Faskowitz et al., 2021). In this study, edges represent stable phase difference between channels/nodes. In the sense of an abstract graph, any pair of nodes can be “connected through” other nodes and edges. However, our edges do not represent paths of information flow, and the synchronous quality of oscillations does not exist between channels separated by more than a single edge. For example, E_*N*_ should not be interpreted as reflecting the shortest paths of communication. Rather, it captures the small-world characteristics of the network. More specifically, that clusters of highly connected nodes may be separated by just one or a few edges. In this way, the combination of E_*N*_ along with our specific edge definition reflects aspects of the network's structural organization. On the other hand, groups of edges capture higher order levels of connectivity. The density of connectivity within highly connected clusters of nodes is described by T_*N*_ while the type of connectivity between clusters is encapsulated by A_*N*_. A clique is subset of *n* network nodes for which every node is directly connected to every other node, and the existence of a clique implies that all *n* channels have a stable phase difference. In Figure 10 we show examples of the real data that generated the A_*N*_ and T_*N*_ values used for trust prediction for one of our participants. Edge distributions for each of the three top performing region-networks are given. We used the Bron–Kerbosch Algorithm (Bron and Kerbosch, 1973) to extract network cliques consisting of three or more channels. Cliques are color coded and noted by *K*_*n*_, where is *n* is the degree. In Figure 10A we see a high degree of connectivity between the central and left frontal channels in *K*_6_. Likewise, Figure 10B shows dense connectivity between the occipital and left parietal channels in both *K*_3_ and *K*_5_. Finally, Figure 10C illustrates substantial connectivity between the parietal and right temporal channel in *K*_7_ as well as the occipital and right temporal channels in *K*_4_ and *K*_5_. The presence of any clique implies that the regions under the EEG electrodes are synchronously integrated and a strong indication of the distributed nature of brain function. Both A_*N*_ and T_*N*_ are greatly impacted by the formation and dissolution of cliques within the network. In turn, both A_*N*_ and T_*N*_ have the greatest impact on trust prediction. Therefore, we see a link between the reorganization of dense clusters of inter-regional brain interaction and trust in an autonomous system.

### 4.1 Limitations

One limitation of this study is the exclusion of EEG data in the delta, theta, and lower alpha bands. This decision was not taken lightly as there is ample evidence that many important characteristics of EEG activity lie in the theta and alpha bands (Hopko and Mehta, 2021). Changes in blink frequency that co-occur with trust report would induce a confound, and we believe the inclusion of data in these band would have biased the results. There were individual variations between both blink frequency, and artifact magnitude among the participants. A major assumption underlying both the feature selection and linear regression methods we utilized is the relative orthogonality between features. Allowing artifacts to remain in the data would have substantially increased the probability of high feature correlations among EEG channels located near the front of the head.

Second, the evolution of trust is dynamic and has been shown to depend on past experience with the system (Rodriguez Rodriguez et al., 2023). Our assumption of a static relationship between trust level and EEG measures represents a first order attempt at modeling what is ultimately a dynamic system. Some of the biggest performance differences were due to the over report of a particular trust level, or the rapid cycling between trust reports. Our assumption was that self-report would occur when participants had adequately assessed the scenario and made a determination of their trust level. It is likely that several participants reported trust while still making the decision about its level. This was apparent in their trust reporting behavior as values would oscillate until finally reaching a stable value. Despite this, there was a relative consensus on which EEG features contained the best predictive information across all participants. However, the ultimate performance of our models appeared to rest on how the participants reported trust. A dynamic model would be better suited at incorporating these variations into trust prediction.

Finally, similar to many studies, our sample size may not have been large enough to capture the variability in EEG characteristics due to age. Future studies should include a larger sample size and participants from more diverse age groups.

### 4.2 Implications and future work

In summary, using an EEG recording device to measure neural activity during a human-autonomy team task, we find that measures of brain integration, rather than the independent activity of individual regions, more effectively capture the cognitive processes that correlate with trust in an autonomous system. We have shown that meaningful patterns may be found within the elicited EEG response, and support the proposition that EEG features can capture cognitive activities that correlate with trust. However, we did not set out to determine if a specific metric, bandwidth, or combination of metrics would generalize as a robust trust measure for our cohort, which would require a significantly broader study. A wider investigation could also explore the trade-off between feature stability and model performance. The removal of certain nodes could have a significant impact on metric values dependent upon their relative importance within the network topology. This type of investigation could only be performed by studying the topological significance of specific nodes using granular measures such as centrality or local efficiency (Yu et al., 2018). For real time estimation of trust, models using network-metrics may be more robust. Myoelectric artifacts will saturate both power and network-metric values, rendering models much less effective. Networks comprised of channels in the posterior part of the head are much less impacted by blinks and other eye and facial movements. Furthermore, a dynamic model of trust estimation would likely capture some of the differences in trust reporting behavior and improve trust prediction accuracy.

Our results indicate that the topology of interactions not only within the frontal lobe, but also between the temporal, parietal and occipital regions are effective at predicting trust in autonomy. In addition, research in the field of cognitive neuroscience has found evidence to suggest that cognitive control capacity may be supported by whole-brain network properties and that dynamic network features may contribute to differences in goal-directed behavior (Nikolaidis and Barbey, 2018). Consequently, the use of network-metrics can provide neuroscientific insight into the mental functions and behaviors that correspond with trust in human-autonomy team tasks (Medaglia et al., 2015). Future investigations could investigate how topological properties of the network change (Bassett et al., 2017; Billings et al., 2021) or identify brain states (O'Connor et al., 2025) that correlate with trust.

## 5 Conclusion

This study investigated the performance of EEG-based models for real time estimation of trust in an autonomous system. We elicited changes in human trust while recording cognitive activity throughout a simulated human-robot team task. Participants were instructed to report changes in trust throughout the experimental trials. We constructed linear regression models to predict changes trust using two types of features derived from the EEG timeseries: (1) Signal-powers over brain regions; (2) EEG inter-channel functional connectivity network-metrics derived from signal phase synchrony. Our results show that measures of neural activity that account for interactions between brain regions more effectively capture the cognitive processes associated with trust than traditional measures of local activity. In addition, features associated with the dynamic reconfiguration of tightly coupled connections between the frontal, parietal, and occipital lobes of the brain had the greatest impact on trust prediction accuracy.

## Data Availability

The raw data supporting the conclusions of this article will be made available by the authors, without undue reservation.

## Footnotes

^1^Capitalization is used to specify a 4 trial section. A GROUP is shown by horizontal bars in Figure 1C

^2^The term channel is derived from the use of the 10–20 International Standard for EEG electrode placement and is commonly used for EEG recording systems.
